# Clinical value of peripheral blood miR-21 and miR-486 combined with CT forearly cancer diagnosis in pulmonary nodulessmoking

**DOI:** 10.1186/s13019-024-03028-8

**Published:** 2024-09-20

**Authors:** Zheng Wang, Jinfeng Liu, Qiang Liu, Yingchun Ren, Qiang Wang, Qing Tian, Zhijie Li, Huining Liu

**Affiliations:** Department of Thoracic Surgery, The First Hospital of Hebei Medical University, No. 89, Donggang Road, Shijiazhuang, 050000 Hebei China

**Keywords:** miR-21 and miR-486, Peripheral blood, Early CT diagnosis, Pulmonary nodules

## Abstract

**Purpose:**

This study aimed to investigate the clinical significance of combining peripheral blood miR-21 and miR-486 with CT for the early cancer diagnosis in pulmonary nodules.

**Methods:**

A total of 215 patients diagnosed with isolated pulmonary nodules with a history of smoking were selected as researchsubjects. 30 healthy volunteers with a history of smoking were recruitedas the control group.The selection of subjectswas based on the presence of isolated pulmonary nodules detected on chest CT scans. The training set consisted of 65 patients with lung nodules and 30 healthy smokers, while the verification setincluded 150 patients with lung nodules.

**Results:**

Compared with the control group, the plasma expression level of miR-210 was significantly higher in the group of patients with benign pulmonary nodules (*P* < 0.05). The level of miR-486-5p was lower in patients with malignant pulmonary nodules compared to those with benign pulmonary nodules (*P* < 0.05). Moreover, the plasma level of miR-210was higher in patients with malignant pulmonary nodules compared to those with benign pulmonary nodules and healthy smokers (*P* < 0.05). The combination of miR-21 and miR-486 yielded an AUC of 0.865, which was significantly higher than any other gene combination (95%CI: 0.653–0.764, *P* < 0.05).

**Conclusions:**

This study offered preliminary evidence supporting the use of peripheral blood miR-21 and miR-486, combined with CT scans, as potential biomarkers for the early cancer diagnosis in lung nodules.

## Introduction

Lung cancer ranks second in both the United States and worldwide as one of the most prevalent cancers, and it is the leading cause of cancer-related deaths [1]. In an effort to detect lung cancer at earlier stages, multiple randomized trials have been conducted in the United States and Europeusing high-resolution CT imaging. The widespread use of CT scans and improved sensitivity has led to a significant increase in the number of solitary pulmonary nodules found in asymptomatic individuals [2]. Solitary pulmonary nodules can be neoplastic (either malignant or benign) or non-neoplastic, and they can be caused by various factors such as benign and malignant tumors, infectious diseases, and abnormal blood vessels in the lungs [3, 4]. However, only a small proportion of solitary pulmonary nodules turn out to be malignant tumors. Hence, it is essential to establish a definite diagnosis of solitary pulmonary nodules before any surgical intervention. This approach enables the early detection of lung cancer, when it is most treatable, and precents unnecessary invasive biopsy and treatment of benign tumors [5]. Although non invasive lung nodule biopsy methods such as puncture biopsy and EBUS-TBLB can provide accurate diagnosis of malignant tumors, they carry inherent risks and may occasionally produce uncertain results. Moreover, a considerable number of locally advanced lung cancer patients in stage III or higher refuse to undergo biopsy due to concerns about tumor implantation expansion during the biopsy process. Conversely, relying solely on a series of chest radiographs can prevent unnecessary surgery for benign conditions, but it may also lead to delays in the accurate diagnosis and treatment of malignant tumors. Therefore, it is of great clinical significance to develop new noninvasive and high-precision diagnostic techniques for lung cancer. One promising method is to identify molecular genetic changes associated with lung cancer in biological fluids [6]. MicroRNAs (miRNAs) play an important role in various biological processes [7–9]. Some miRNAs act as oncogenes or tumor suppressorsduring tumor development, and their altered expression levels can contribute to tumor initiation and progression. Abnormal miRNAs expression patterns in lung cancer have been proposed as potential biomarkers. This distinct expression patterns of miRNA in tumor cells compared to normal cellscan be detected in blood and tissuesamples [10, 11]. By analyzing miRNA profilesin individuals with and without lung cancer, researchers can identify specific signatures or characteristics of miRNA associated with the disease [12, 13]. Plasma miRNA has emerged as a promising circulating biomarker [14]. Abnormal miRNA expression in plasma can differentiate individuals with lung cancer from those without the disease. Therefore, the aim of this study was to investigate whether plasma miRNA could serve as a potential biomarker of lung cancer in individuals with solitary pulmonary nodules detected by CT. Theresults of this study demonstrated that a combination of miR-21 and miR-486 might serve as a circulating biomarker for distinguishing lung tumors from benign solitary pulmonary nodules.

### Participants

From January 2019 to December 2022, we enrolled 215 patients diagnosed with isolated pulmonary nodules with a history of smoking at our hospital. The age range of theparticipantswas 48 to 76 years, with an average age of 66.42 ± 6.82 years. Among them, there were130 males and 85 females. Additionally, 30 healthy volunteers with a history of smoking were included as the control group.The selection of participants was based on the presence of solitary pulmonary nodules detected through chest CT scans. Prior to their inclusion in the study, all patients provided informed consent. Among the participants, 65individuals with pulmonary nodules and 30 healthy volunteers were assigned to the training set, while the remaining 150 patients with pulmonary nodules were assigned to the verification set.

#### Inclusion criteria

The inclusion criteria for the study were as follows: patients diagnosed with isolated pulmonary nodules with a history of smoking; presence of single, round/nearly round lung lesions identified in the chest CT scans; nodule diameter ≤ 3 cm; absence of noticeable symptoms; and confirmation of the final diagnosis samples obtained via CT-guided transthoracic biopsy, transbronchial biopsy, assisted thoracoscopic surgery, or surgical resection.

#### Exclusion criteria

The exclusion criteria for the study were: individuals previously diagnosed with benign or malignant pulmonary nodules; incomplete clinical data; and patients who refused to provide informed consent.

#### Medical ethics considerations

This study was allowed by the local institutions review committee and conducted in accordance with the principles in the Helsinki Declaration.

## Materials and methods

### Plasma sample collection

Peripheral blood samples were collected using a standardized blood drawing procedure. A total of 10 ml of blood was drawn into a BD vacuum spray-coated K2EDTA tube. The blood samples were thenprocessed within 2 h of collection by centrifugation. The plasma was carefully transferred to a test tube and stored at-80 ℃ until further use.

### Isolation of RNA

RNA isolation from plasma was performed using the mirVana miRNA isolation kit, following our previous research protocols. The purity and concentration of the extracted RNA were assessed using a double-beam ultraviolet spectrophotometer. The integrity of the RNA samples was evaluated using a bioanalyzer 2100. Only RNA samples with a RNA integrity value greater then8 were included for further analysis.

### qRT-PCR

qRT-PCR was performed using the TaqMan MicroRNA RT Kit. The expression level of five specific miRNAs (miR-21, miR-126, miR-210, miR-375, and miR-486) in plasma were quantified using the Ct method and the 2^−ΔΔCt^formula. The Ct value of the target miRNA was normalized to the Ct value of miR-16.

### Statistical analysis

All data in this study were analyzed using SPSS20.0 statistical analysis software (IBM, USA). The measurement data were presented as mean ± standard deviation ($\bar x \pm \;{\rm{s}}$). Groups comparisons were performed using either one-way ANOVA or repeated measurement ANOVA, followed by LSD-t test for intergroup comparisons. The counting data were presented as percentage (%), and group comparisons groups were analyzed using the χ^2^ test. A *P* < 0.05 was considered statistically significant.

## Results

### Analysis of clinical characteristics of subjects

The control group had an average age of 65.38 ± 5.24 years, consisting of 18 males and 12 females, with an average BMI of 23.54 ± 2.05 kg/m^2^. The benign group had an average age of 67.51 ± 6.38 years, including 21 males and 13 females, with an average BMI of 22.39 ± 1.88 kg/m^2^. The malignant group had an average age of 66.42 ± 4.75 years, comprising 20 males and 11 females, with an average BMI of 23.56 ± 2.17 kg/m^2^. In terms of NSCLC staging, there were 12 cases in stage I, 8 cases in stage II, and 11 cases in stage III-IV. Among the cases, 17 cases (54.83%) were diagnosed as adenocarcinoma, and 45.17% were squamous cell carcinoma (Table 1).

**Table 1:** Analysis of the clinical data of patients in the training set ($\bar x \pm \;{\rm{s}}$)

Index	Control group (*n* = 30)	Benign lung nodule group (*n* = 34)	Malignant pulmonary nodule group (*n* = 31)
Age (years)	65.38 ± 5.24	67.51 ± 6.38	66.42 ± 4.75
Gender (male/female)	18/12	21/13	20/11
BMI(kg/m^2^)	23.54 ± 2.05	22.39 ± 1.88	23.56 ± 2.17
Nodule size (cm)	-	1.77 ± 0.24	2.65 ± 0.35
NSCLC staging	-	-	
I	-	-	12(38.71%)
II	-	-	8(25.81%)
III-IV	-	-	11(35.48%)
Glandular cancer	-	-	17(54.83%)
Squamous cell carcinoma	-	-	14(45.17%)
Benign inducement	-	-	-
Inflammatory lesion	-	23(67.64%)	-
Meat quality	-	3(8.82%)	-
Granuloma	-	3(8.82%)	-
Fibrosis	-	2(14.72%)	-

### Analysis of the clinical characteristics of patients in the validation set

Statistical analysis of the general data for patients in the verification set revealed that the benign pulmonary nodules group had an average age of 65.29 ± 5.62 years, including 48 males and 32 females, with an average BMI of 22.54 ± 1.76 kg/m^2^ and an average nodule size of 1.62 ± 0.30 cm. The majority of nodules in this group were inflammatory nodules (63.75%). The malignant group in the verification set had an average age of 67.68 ± 4.41 years, containing 37 males and 23 females, with an average BMI of 23.32 ± 1.95 kg/m^2^. The average nodule size was 2.71 ± 0.36 cm, with inflammatory pulmonary nodules being the most common (67.64%). In terms of NSCLC staging, there were 22 cases in stage I, 29 cases in stage II, and 19 cases in stage III-IV. Among these cases, 37 (52.85%) were diagnosed as adenocarcinoma, and 33 (47.15%) were squamous cell carcinoma (Table 2).

**Table 2:** Analysis of the clinical features of patients in the verification set ($\bar x \pm \;{\rm{s}}$)

Index	Benign lung nodule group (*n* = 80)	Malignant pulmonary nodule group (*n* = 70)
Age (years)	65.29 ± 5.62	67.68 ± 4.41
Gender (male/female)	48/32	37/23
BMI(kg/m2)	22.54 ± 1.76	23.32 ± 1.95
Nodule size (cm)	1.62 ± 0.30	2.71 ± 0.36
NSCLC staging		
I	-	22(31.42%)
II	-	29(41.42%)
III-IV	-	19(27.14%)
Glandular cancer	-	37(52.85%)
Squamous cell carcinoma	-	33(47.15%)
Benign inducement		
Inflammatory lesion	51(63.75%)	-
Meat quality	7(8.75%)	-
Granuloma	8(10.00%)	-
Fibrosis	6(7.50%)	-

### Analysis of abnormal miRNA expression in the training set

**Fig. 1:**
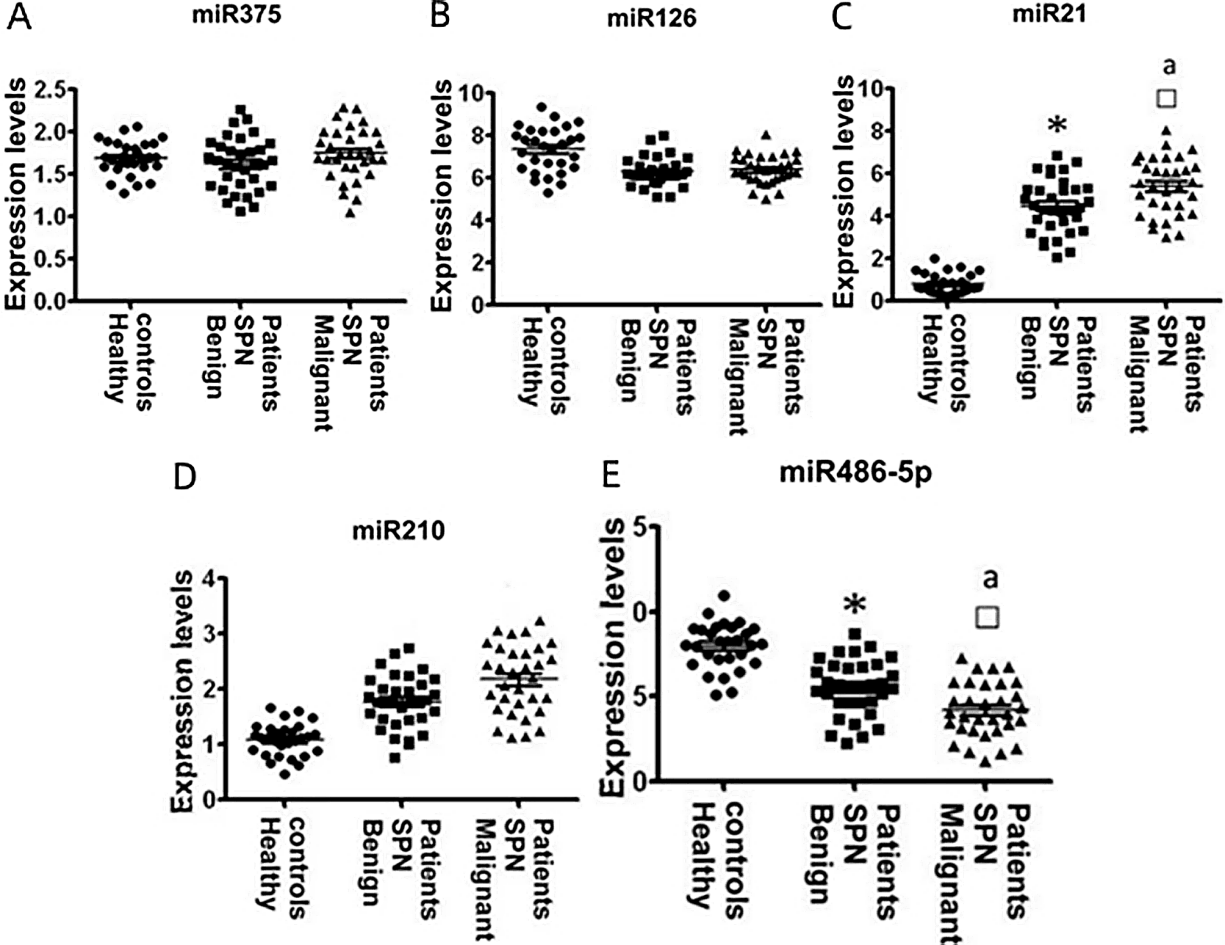
Analysis of abnormal miRNA expression in training subjects

Abnormal miRNA expression in plasma was determined using qRT-PCR. There were no statistically significant differences observed in the expression levels of miR-126 and miR-375 among the three groups (*P* > 0.05).However, comparison with the control group, the plasma expression level of miR-210 was significantly increased in patients with benign pulmonary nodules (*P* < 0.05). Furthermore, when compared to individuals with benign pulmonary nodules, those with malignant pulmonary nodules exhibited higher expressionlevels of plasma miR-210 (*P* < 0.05). Conversely, the expression of plasma miR-486-5p was significantly decreased in individuals with benign pulmonary nodules compared to the control group, (*P* < 0.05). Additionally, the patients with malignant pulmonary nodules displayed lower expression levels of miR-486-5p in plasma compared to those with benign pulmonary nodules (*P* < 0.05). To sum up, the plasma expression level of miR-210 inpatients with malignant pulmonary nodules was greatly higher than that in patients with benign pulmonary nodules and healthy smokers (*P* < 0.05). Similarly, the plasma expression level of miR-486-5p in patients with malignant pulmonary nodules was lower than that in patients with benign pulmonary nodules and healthy smokers. Therefore, these two miRNAs were considered to have the potential to serves asplasma biomarkers for differentiating malignant pulmonary nodules (Fig. 1; Table 3).

**Table 3:** Analysis of abnormal miRNAexpression in the training set ($\bar x \pm \;{\rm{s}}$)

Gene	Control group (*n* = 30)	Benign lung nodule group (*n* = 34)	Malignant pulmonary nodule group (*n* = 31)	Variance ratio	*P* value
miR-375	1.72 ± 0.34	1.64 ± 0.31	1.71 ± 0.30	5.227	0.144
miR-126	7.49 ± 1.03	6.37 ± 0.58	6.42 ± 0.54	3.449	0.295
miR-21	0.65 ± 0.18	4.28 ± 0.85	5.66 ± 0.72	14.056	0.007
miR-210	1.05 ± 0.25	1.81 ± 0.34	1.15 ± 0.54	4.672	0.142
miR-486	7.94 ± 0.77	5.42 ± 0.55	4.14 ± 0.32	12.005	0.013

### The relationship between plasma miRNA expression and clinical features

Pearson correlation analysis revealed that the expression changes of the miR-21 and miR-486-5p genes were not associated with age, sex, or histological pattern of the participants (*P* > 0.05). However, these expression changes were significantly correlated with smoking (*P* < 0.05). In addition, the expression levels of miRNA in plasma were found to be related to the size of pulmonary nodules (*P* < 0.05) (Table 4).

**Table 4:** Relationship between plasma miRNA expression and clinical features (S)

Gene	Age	Sex	Smoking	Nodular size	NSCLC staging
miR-375	0.083(0.336)	-0.114(0.319)	0.527(0.003)	0.2369(0.074)	-0.005(0.073)
miR-126	-0.028(0.168)	-0.031(0.332)	0.806(0.001)	0.029(0.068)	0.313(0.08)
miR-21	0.076(0.024)	-0.0639(0.509)	0.21(0.024)	0.081(0.038)	-0.124(0.06)
miR-210	-0.032(0.556)	-0.234(0.274)	0.75(0.017)	0.213(0.215)	-0.044(0.319)
miR-486	0.159(0.112)	-0.136(0.114)	0.25(0.003)	0.135(0.021)	-0.046(0.537)

### ROC curve analysis of plasma miRNA expression levels

**Fig. 2:**
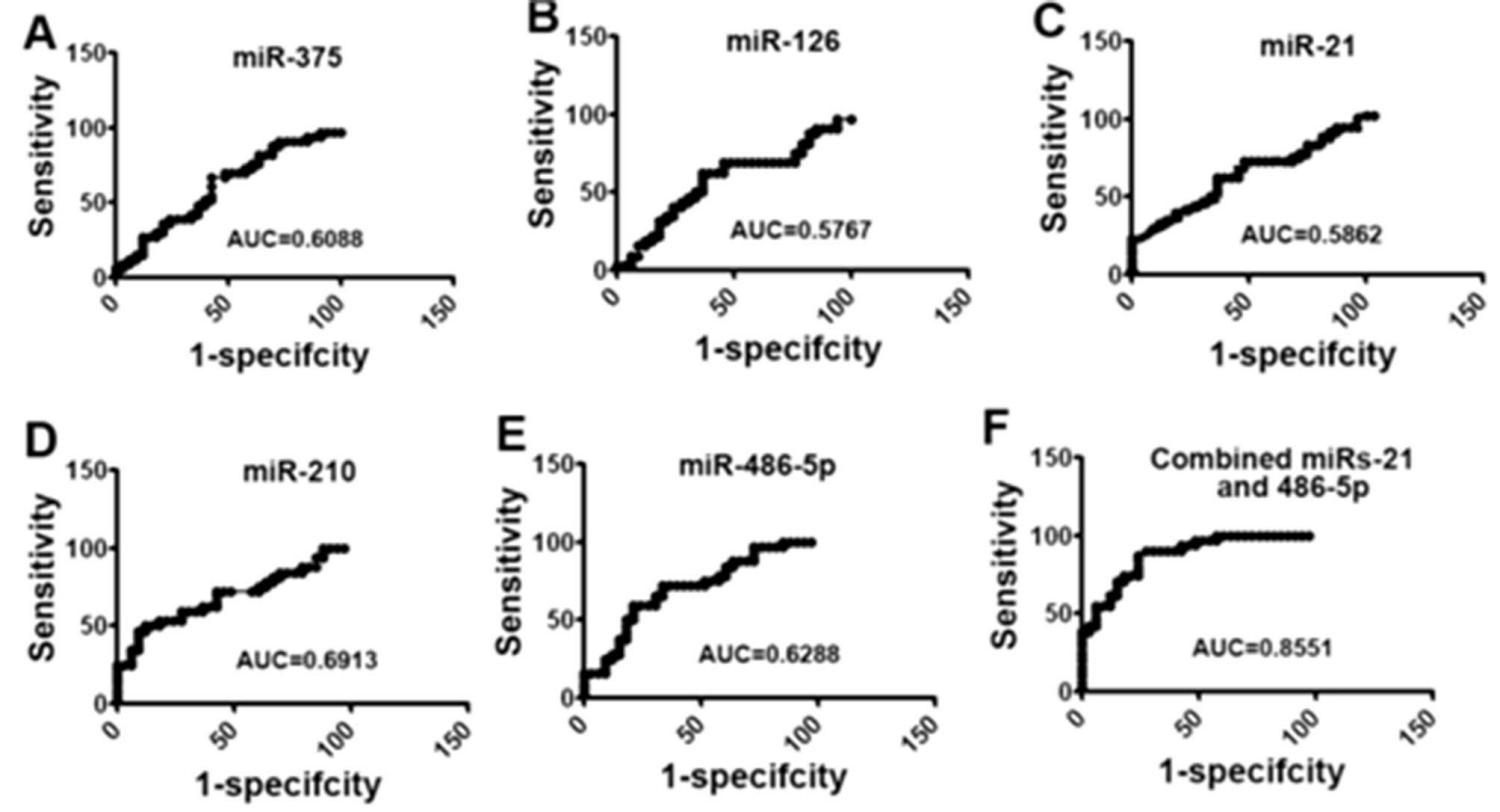
ROC curve of plasma miRNA expression levels

TheAUC values demonstrated that miRNA analysis provided accurate sensitivity and specificity in distinguishing between benign nodules and malignant nodules. Figure 2A-E depicted the ROC curves of five individual miRNAs, while Fig. 2F displayed the ROC curve of the combined miR-210 and miR-486-5p.

### Wilcoxon rank sum test

The AUC for the combination of miR-21 and miR-486 was 0.865, which was significantly higher than any other combination of genes (95% CI: 0.653–0.764, *P* < 0.05) (Table 5).

**Table 5:** Wilcoxon rank sum test ($\bar x \pm \;{\rm{s}}$)

ROC model	zone	standard deviation	95% confidence interval
MiR-21 and miR-210	0.724	0.052	0.352–0.885
MiR-21 and miR-486	0.855	0.016	0.813–0.942
MiR-20 and miR-486	0.726	0.057	0.653–0.764
MiR-20, miR-21 and miR-486	0.773	0.054	0.781–0.966

### Evaluation and analysis of miRNA in the validation set

The diagnostic performance of miR-21 combined with miR-486 in pulmonary nodules was evaluatedthrough sensitivity and specificity analysis. The specificity and sensitivity of the combination of miR-21 and miR-486 in differentiating pulmonary nodules were 84.37% and 77.29%, respectively. There were no statistical differences in the diagnostic sensitivity and specificity of miR-21 and miR-486 concerning lung tumor staging and tumor tissue (*P* > 0.05). These findingsdiscovered that miR-21 and miR-486 could be used as reliable markers forearly-stage lung tumors in individuals detected through CT scans (Table 6).

**Table 6:** Evaluation and analysis of miRNA in the validation set ($\bar x \pm \;{\rm{s}}$)

Index	Specificity	Sensitivity
All subjects	84.37%	77.29%
Histological types		
glandular cancer	84.37%	75.33%
Squamous cell carcinoma	84.37%	76.49%
Stages		
I	84.37%	75.00%
II	84.37%	79.21%
III	84.37%	73.48%

## Discussion

When solitary pulmonary nodules are detected in the lungs, CT scans are commonly used by doctors to evaluate their nature and possible causes [15]. Solitary pulmonary nodules refer to small, single lump or nodules in lung tissue that are typically less than 3 cm in diameter. These nodules appear as round or oval shapes on CT images and are clearly distinguishable from the surrounding lung tissue [16]. CT examination, a widely used non-invasive imaging technique, utilizes X-ray and computer technology to provide high-resolution lung images. It enables detailed assessment of structural information such as size, shape, density, and other characteristics of lung nodules. Through CT scans, doctors can quantitatively and qualitatively evaluate solitary pulmonary nodules to determine whether they are benign or malignant [17]. In recent years, miRNA has gained significant research interest as an important molecule in gene expressionregulation [18]. MiRNA is a type of noncoding RNA molecule about 22 nucleotides in length. It can bind to the mRNA of the target genes, thereby inhibiting or promoting their transcription and translation processes. Abnormal miRNA expression is associated with the development of various cancers. In the study of solitary pulmonary nodules, miRNA has emerged as a potential biomarker of interest. By analyzing the expression levels of miRNA in lung tissue or peripheral blood, specific miRNA patterns related to pulmonary nodules can be identified [19]. The abnormal expression of these miRNA may be linked to the occurrence, progression, and differentiation between benign and malignant pulmonary nodules. Hence, researchers have begun exploring the potential of miRNA as a diagnostic tool to aid in the identification of cancer in solitary pulmonary nodules. The combination of CT examination and miRNA analysis has the potential to enhance the accuracy of early diagnosis of solitary pulmonary nodules [20]. CT provides morphological features of pulmonary nodules, while miRNA analysis offers molecular-level biomarker information. By integrating these two methods, the benign and malignant potential of solitary pulmonary nodules can be better evaluated, thereby guiding subsequent treatment decisions.

As a significant factor in tumor development, the up-regulation of miR-21 has been associated with tumor occurrence and progression, making it a potential biomarker for tumors [21]. For instance, recent research has identified miR-21 as one of the plasma miRNA scapable of distinguishing patients with early-stage lung cancer. Moreover, miR-486-5p is often under-expressed in various tumors [22]. In this study, the expressionlevels of miR-486-5p were found to be lower in lung tumor tissues compared to matched normal lung tissues. Consistent with previous findings in tissue samples, this study demonstrated that miR-486-5p expressionin the plasma of lung cancer patients was lower thanin those with benign and healthy smokers. The results from surgical tissues and plasma samples suggested that down-regulation of miR-486-5p may play a role in tumor inhibition during lung cancer development. Several laboratories have investigated the diagnosis of lung cancer by analyzing cancer-related miRNA in blood [23, 24]. For example, a recent study identified specific miRNA profiles in plasma that could be used for the diagnosis of lung cancer and the detection of t invasive diseases even before spiral CT imaging [25]. Considering the accuracy, the combined use of miRNAs and CT in the future may overcome conquer the low sensitivity limitation of imaging analysis in the early detection of lung cancer.

We aimed toassess the clinical value of peripheral blood miR-21 and miR-486 in conjunction with CT scans for the early diagnosis of cancer in pulmonary nodules. The study included 215 patients diagnosed with solitary pulmonary nodules and 30 healthy volunteers with a history of smoking as the control group. It was found that the plasma expression level of miR-21 increased in the benign and malignant groups compared to thecontrol group, while the expression level of miR-486-5p decreased. Furthermore, the expression level of miR-21 in the malignant group was significantly higher than that in the benign group and control group, whereas the expressionlevel of miR-486-5p in the malignant group was lower thanin the benign group and control group. The study also found that the expression levels of these two miRNAs were linked to smoking and the size of pulmonary nodules. The combination of miR-21 and miR-486 exertedhigh accuracy in distinguishing the nature of pulmonary nodules, with an AUC of 0.855, significantly higher than other gene combinations. The combined use of miR-21 and miR-486 exhibited a sensitivity of 77.29% and a specificity of 84.37%. In addition, there were no statistical differences between miR-21 and miR-486 in diagnostic sensitivity and specificity for lung tumor staging and tumor tissue.

Our study highlighted the potential of combining peripheral blood miR-21 and miR-486 with CT scans as biomarkers for the early diagnosis of cancer in pulmonary nodules. The abnormal expression of miR-21 and miR-486 was found to be related to malignant lung nodules, and their combined use couldenhancediagnosisaccuracy. However, further research is necessary to verify these findings and explore their potential applications in clinical practice. It is important to acknowledge the limitations of this study. First of all, the research sample size was relatively small, which may introduce selection bias. Secondly, the study only included patients from a single hospital, which may limit the generalizability of the findings to otherregions. Thirdly, this study focused solely on the expression levels of miR-21 and miR-486, potentially overlooking the role of other miRNAs. Therefore, further large-scale, multi-center studies are needed.

In conclusion, this study providedinitial evidence supporting the potential of miR-21 and miR-486 in peripheral blood, combined with CT scans, as biomarkers for the early cancer diagnosis in pulmonary nodules. These findings offered new insights into the development of early diagnosis of lung cancer and held promise for their future clinical application.

## Data Availability

The datasets analyzed during the current study are not publicly available due to the personal privacy but are available from the corresponding author on reasonable request.

## Abbreviations

MiRNAs: MicroRNAs

## References

[1] Guo X, Jia X, Zhang D, et al. Indeterminate pulmonary subsolid nodules in patients with no history of cancer: growing prediction, CT pattern, and pathological diagnosis[J]. DiagnIntervRadiol. 2022;28:230–8. 10.5152/dir.2022.211100 PMC9634916 35748205

[2] Chen D, Yan Y, Wang X, et al. Chronic alcohol exposure promotes HCC stemness and metastasis through β-catenin/miR-22-3p/TET2 axis[J]. Aging. 2021;13:14433–55. 34019487 10.18632/aging.203059 PMC8202861

[3] Marciano BE, Olivier KN, Folio LR, et al. Pulmonary manifestations of GATA2 Deficiency[J]. Chest. 2021;160:1350–9. 34089740 10.1016/j.chest.2021.05.046 PMC8546236

[4] Chen R, Zhang C, Cheng Y, et al. LncRNA UCC promotes epithelial-mesenchymal transition via the miR-143-3p/SOX5 axis in non-small-cell lung cancer[J]. Lab Invest. 2021;101:1153–65. 33824420 10.1038/s41374-021-00586-6

[5] Ge N, Mao C, Yang Q, et al. Single nucleotide polymorphism rs3746444 in miR-499a affects susceptibility to nonsmall cell lung carcinoma by regulating the expression of CD200[J]. Int J Mol Med. 2019;43:2221–9. 30864695 10.3892/ijmm.2019.4124

[6] Kim RY, Oke JL, Pickup LC, et al. Artificial Intelligence Tool for Assessment of Indeterminate Pulmonary nodules detected with CT[J]. Radiology. 2022;304:683–91. 35608444 10.1148/radiol.212182 PMC9434821

[7] Almquist DR, Ernani V, Sonbol MB. Diffuse idiopathic pulmonary neuroendocrine cell hyperplasia: DIPNECH[J]. CurrOpinPulm Med. 2021;27:255–61. 10.1097/MCP.0000000000000776 33927131

[8] Liu M, Zhou Z, Liu F, et al. CT and CEA-based machine learning model for predicting malignant pulmonary nodules[J]. Cancer Sci. 2022;113:4363–73. 36056603 10.1111/cas.15561 PMC9746043

[9] Gilbert FJ, Harris S, Miles KA, et al. Dynamic contrast-enhanced CT compared with positron emission tomography CT to characterise solitary pulmonary nodules: the SPUtNIk diagnostic accuracy study and economic modelling[J]. Health Technol Assess. 2022;26:1–180. 35289267 10.3310/WCEI8321

[10] Gao S, Guo W, Liu T, et al. Plasma extracellular vesicle microRNA profiling and the identification of a diagnostic signature for stage I lung adenocarcinoma[J]. Cancer Sci. 2022;113:648–59. 34837453 10.1111/cas.15222 PMC8819331

[11] Araujo-Filho JAB, Halpenny D, McQuade C, et al. Management of pulmonary nodules in oncologic patients: AJR Expert Panel Narrative Review[J]. AJR Am J Roentgenol. 2021;216:1423–31. 33355489 10.2214/AJR.20.24907

[12] Gheysens G, De Wever W, Cockmartin L, et al. Detection of pulmonary nodules with scoutless fixed-dose ultra-low-dose CT: a prospective study[J]. EurRadiol. 2022;32:4437–45. 10.1007/s00330-022-08584-y 35238969

[13] Hu B, Ren W, Feng Z, et al. Correlation between CT imaging characteristics and pathological diagnosis for subcentimeter pulmonary nodules[J]. Thorac Cancer. 2022;13:1067–75. 35212152 10.1111/1759-7714.14363 PMC8977167

[14] Liu J, Zhao L, Han X, et al. Estimation of malignancy of pulmonary nodules at CT scans: Effect of computer-aided diagnosis on diagnostic performance of radiologists[J]. Asia Pac J Clin Oncol. 2021;17:216–21. 32757455 10.1111/ajco.13362

[15] He Y, Ren S, Wang Y, et al. Serum microRNAs improving the diagnostic accuracy in lung cancer presenting with pulmonary nodules[J]. J Thorac Dis. 2018;10:5080–5. 30233883 10.21037/jtd.2018.07.138 PMC6129919

[16] Yang G, Wang T, Qu X, et al. Exosomal miR-21/Let-7a ratio distinguishes non-small cell lung cancer from benign pulmonary diseases[J]. Asia Pac J Clin Oncol. 2020;16:280–6. 32525285 10.1111/ajco.13343 PMC7496917

[17] Tao R, Cao W, Zhu F, et al. Liquid biopsies to distinguish malignant from benign pulmonary nodules[J]. Thorac Cancer. 2021;12:1647–55. 33960710 10.1111/1759-7714.13982 PMC8169297

[18] Wang YZ, Lv YB, Li GY, et al. Value of low-dose spiral CT combined with circulating miR-200b and miR-200c examinations for lung cancer screening in physical examination population[J]. Eur Rev Med Pharmacol Sci. 2021;25:6123–30. 34661272 10.26355/eurrev_202110_26890

[19] Chen F, Liu YB, Fu BJ, et al. Clinical and computed tomography (CT) characteristics of pulmonary nodules caused by cryptococcal Infection[J]. Infect Drug Resist. 2021;14:4227–35. 34703249 10.2147/IDR.S330159 PMC8523807

[20] Gao Y, Hua M, Lv J, et al. Reproducibility of radiomic features of pulmonary nodules between low-dose CT and conventional-dose CT[J]. Quant Imaging Med Surg. 2022;12:2368–77. 35371962 10.21037/qims-21-609 PMC8923849

[21] Venkadesh KV, Setio AAA, Schreuder A, et al. Deep learning for Malignancy Risk Estimation of Pulmonary nodules detected at low-dose screening CT[J]. Radiology. 2021;300:438–47. 34003056 10.1148/radiol.2021204433

[22] Bartlett EC, Kemp SV, Rawal B, et al. Defining growth in small pulmonary nodules using volumetry: results from a coffee-break CT study and implications for current nodule management guidelines[J]. EurRadiol. 2022;32:1912–20. 10.1007/s00330-021-08302-0 PMC8831344 34580748

[23] Wu Z, Wang F, Cao W, et al. Lung cancer risk prediction models based on pulmonary nodules: a systematic review[J]. Thorac Cancer. 2022;13:664–77. 35137543 10.1111/1759-7714.14333 PMC8888150

[24] Patel N, Xu W, Deng Y, et al. Cross-scale Integration of Nano-Sized Extracellular vesicle-based biomarker and Radiomics features for Predicting suspected Sub-solid Pulmonary Nodules[J]. J Biomed Nanotechnol. 2021;17:1109–22. 34167625 10.1166/jbn.2021.3097

[25] Farjah F, Monsell SE, Smith-Bindman R, et al. Fleischner society guideline recommendations for incidentally detected pulmonary nodules and the probability of Lung Cancer[J]. J Am CollRadiol. 2022;19:1226–35. 10.1016/j.jacr.2022.06.018 36049538

